# Neuropsychological evaluation of cognitive decline in Latin America

**DOI:** 10.1002/alz70857_101769

**Published:** 2025-12-25

**Authors:** Maira Okada de Oliveira

**Affiliations:** ^1^ Global Brain Health Institute (GBHI), University of California San Francisco (UCSF); & Trinity College Dublin, San Francisco, CA, USA; Cognitive Neurology and Behavioral Unit (GNCC), University of Sao Paulo, Sao Paulo, Sao Paulo, Brazil

## Abstract

**Background:**

Investigating cognitive decline remains a challenge, especially due to regional differences. Latin American countries face disparities in access to healthcare, diagnostic tools, and treatments. Clinicians should actively inquire about cognitive decline complaints in patients, particularly those over 60 years of age. Cognitive and functional testing is crucial for evaluating patients with these complaints.

**Method:**

The implementation of quick, accessible, and easily scored screening tools that are less influenced by cultural and educational factors is recommended.

**Result:**

When cognitive decline is suspected, screening tools such as the 10‐Point Cognitive Screener (10‐CS) for patients and the Cognitive Change Questionnaire (QMC8) for caregivers are suggested; ideally, both should be administered. Additional cognitive assessment tools recommended for diagnosing mild cognitive impairment (MCI) and dementia include the Mini‐Mental State Examination (MMSE), Montreal Cognitive Assessment (MoCA), Addenbrooke's Cognitive Examination‐Revised/III (ACE‐R or ACE‐III), Rowland Dementia Assessment Scale (RUDAS), and Brief Cognitive Screening Battery (BCSB). Functional assessment tools, such as the Pfeffer Functional Activities Questionnaire (FAQ), should also be considered.

If concerning results are observed, further clinical investigation is advised, including differential diagnoses and the exclusion of reversible causes. Regular patient follow‐ups are essential, with cognitive status reassessed every six months to monitor changes and educate patients on modifiable risk factors for dementia prevention.

**Conclusion:**

In this presentation, will be provide a recommendation on best practices in detecting cognitive decline with an adaptation to the Latin American culture and combination of available resources.

